# Bioinspired Laser-Textured Aluminum Surfaces for Anti-Icing: Coupled Effects of Hydrophobic Coating Chemistry and Surface Morphology

**DOI:** 10.3390/biomimetics11080585

**Published:** 2026-08-17

**Authors:** Borut Gregorčič, Armin Hadžić, Jure Berce, Matevž Zupančič, Matic Može, Iztok Golobič

**Affiliations:** 1 Faculty of Mechanical Engineering, University of Ljubljana, Aškerčeva cesta 6, SI-1000 Ljubljana, Slovenia; bg07808@student.uni-lj.si (B.G.); armin.hadzic@fs.uni-lj.si (A.H.); jure.berce@fs.uni-lj.si (J.B.); matevz.zupancic@fs.uni-lj.si (M.Z.); iztok.golobic@fs.uni-lj.si (I.G.); 2 KRKA, d.d., Novo mesto, Šmarješka cesta 6, SI-8501 Novo Mesto, Slovenia

**Keywords:** bioinspired surfaces, biomimetic superhydrophobicity, ice adhesion, freezing delay, laser texturing, aluminum, hydrophobic coatings, wetting-state stability

## Abstract

Natural water-repellent surfaces use hierarchical texture and low surface energy to minimize liquid adhesion, and this principle has inspired engineered superhydrophobic surfaces for passive anti-icing. However, whether such bioinspired water-repellent architectures remain beneficial during freezing and ice detachment depends on the stability of the wetting state and on the interaction between surface texture and coating chemistry. This study evaluates the anti-icing performance of smooth and laser-textured 1050A aluminum surfaces functionalized with different hydrophobic agents. Freezing delay measurements at −18 °C and ice adhesion strength measurements at −20 °C were conducted, together with wettability, surface free energy, roughness, and morphology analyses, to compare different coatings on identical morphologies and to isolate the effect of laser-generated texture for the same coating chemistry. On smooth surfaces, fluorinated alkyl phosphonic acid coating provided the largest reduction in ice adhesion strength, decreasing it by approximately 72% relative to the non-functionalized reference, while alkyl phosphonic acid coating reduced it by approximately 50%. In contrast, polydimethylsiloxane showed the longest freezing delay, with a mean value of 907 s, whereas the fatty acid-based coatings exhibited shorter freezing delays than the bare reference surface. On laser-textured surfaces, all coatings initially produced highly water-repellent wetting states. However, the differences in ice adhesion strength were markedly reduced and no longer followed the same ranking as on smooth surfaces. Polydimethylsiloxane again exhibited the most favorable freezing delay, while fluorinated alkyl phosphonic acid showed the poorest performance on the textured substrate. These results show that the bioinspired superhydrophobic state created by laser texturing does not by itself guarantee improved anti-icing performance, as under icing conditions, texture-mediated wetting, local liquid penetration, condensation or frost formation inside the texture, and mechanical interlocking can dominate over the nominal low-surface-energy chemistry.

## 1. Introduction

Ice accretion on exposed engineering surfaces remains a major reliability and safety concern in aviation [1,2], wind energy [3], power transmission [4], transportation [5,6], and outdoor infrastructure because it increases aerodynamic drag and structural load, blocks flow passages, and promotes vibration and fatigue [7]. Consequently, there is sustained interest in passive icephobic approaches that can either delay ice formation or enable ice removal with low mechanical or thermal input, thereby reducing the operational disadvantage of conventional de-icing approaches [8].

Biological surfaces often control water, ice, and contaminant interactions through the coupling of hierarchical topography and surface chemistry. Lotus leaves, rice leaves, insect wings, springtail cuticles, and water strider legs are widely discussed examples of natural interfaces in which micro/nanoscale structures reduce the effective liquid–solid contact area, stabilize air pockets, and promote droplet mobility. These natural design principles have motivated the development of bioinspired superhydrophobic surfaces for engineering applications, including passive anti-icing, where rapid droplet shedding and reduced water–solid contact can delay freezing or reduce the probability of ice formation.

Nevertheless, transferring natural water repellency to icephobic engineering surfaces is not straightforward. Under condensation or frost-forming conditions, water can penetrate the surface texture, replace trapped air, and freeze within the micro-/nanoscale features. This can increase contact-angle hysteresis, promote mechanical interlocking, and consequently increase ice adhesion. Therefore, bioinspired surfaces intended for anti-icing applications must be evaluated not only through static contact angle but also through freezing delay, ice adhesion strength, droplet mobility, and post-icing surface stability.

The anti-icing performance of a surface is commonly assessed through two complementary metrics. The first is the freezing delay of a supercooled droplet, which reflects the combined effects of heat transfer and heterogeneous nucleation on the time to freezing [9]. The second is the ice adhesion strength, most commonly reported as the shear stress required to detach an ice block from the surface, which determines whether formed ice can be removed by aerodynamic loads or by low-intensity mechanical actuation [10,11]. In addition to these two metrics, water repellency is also an important aspect of anti-icing performance because it determines whether a droplet can roll off or detach from the surface before sufficient cooling and freezing occur. Importantly, these aspects are not necessarily correlated because the freezing pathway can change the dominant interfacial failure mechanism. Direct droplet freezing can produce relatively weak interfaces on some water-repellent surfaces, whereas condensation and frost growth within surface textures can generate mechanical fixing and substantially increase the measured adhesion [12]. Accordingly, superhydrophobicity does not necessarily imply icephobicity. Nosonovsky and Hejazi [13] emphasized the importance of contact-angle hysteresis and interfacial fracture, while Starostin et al. [14] demonstrated that robust anti-icing performance can be achieved by combining hierarchical morphology with low-surface-energy chemistry and a stable Cassie wetting state. These studies confirm that apparent static contact angle alone is insufficient to predict icephobic performance. Surface engineering strategies aimed at lowering ice adhesion and extending freezing delay often rely on controlling the real contact area between ice and the solid, reducing pinning of the three-phase contact line prior to freezing, and promoting interfacial crack initiation during ice removal [15]. Superhydrophobic surfaces are frequently investigated because water repellency can reduce droplet residence time, limit the extent of liquid–solid contact, and delay nucleation under specific conditions [16,17]. Nevertheless, many studies report large scatter in ice adhesion for nominally similar water contact angles because wetting state stability during icing is strongly affected by texture geometry, ambient humidity, and the formation of frost or liquid bridges within surface features [18,19].

Laser surface texturing has therefore emerged as a particularly attractive method for icephobic surface development because it enables controlled micro- and nanoscale topographies to be created directly on structural metals and selected polymers, with strong bonding to the substrate and good repeatability [20,21]. Laser texturing is generally combined with a low-surface-energy modification layer, most commonly fluorinated silanes [22,23], fluorinated phosphonic acids [24,25], or thin polymeric coatings [26,27], to obtain high water repellency while retaining the mechanical robustness of the underlying substrate.

From a broader perspective, such laser-textured water-repellent surfaces can also be regarded as biomimetic because they follow the same general design principle observed in natural liquid-repellent interfaces, namely the combination of surface texture and low interfacial affinity to reduce liquid adhesion and promote droplet mobility. Classical examples include lotus leaves, which exhibit superhydrophobic self-cleaning behavior, and springtail cuticles, whose surface architecture has inspired the design of highly liquid-repellent engineered surfaces. In the anti-icing field, such biomimetic concepts are increasingly explored to design surfaces that delay freezing and reduce ice adhesion by promoting water shedding and limiting liquid penetration into the texture. Depending on the specific texture geometry and the resulting wetting state during icing, laser-textured surfaces of this type have been shown to reduce ice adhesion from tens of kilopascals to only a few kilopascals, and in some cases to values approaching the lower measurement limits reported for dry surfaces.

Recent work increasingly focuses on the mechanisms that govern long-term performance under realistic conditions. These include repeated icing and de-icing cycles, the presence of humidity that promotes condensation within surface features, and the progressive transition from an air-retaining interface to a fully wetted interface that enables mechanical interlocking of ice [28]. In parallel, multifunctional concepts have been proposed, including photothermal laser-textured surfaces designed to suppress icing by local temperature elevation during illumination and to stabilize low ice adhesion during cycling [29]. From a biomimetic design perspective, these studies also illustrate a central unresolved question: whether the texture-induced air retention that enables water repellency can remain stable under the colder, more humid, and mechanically disruptive conditions associated with icing and de-icing. The main outcomes of representative laser-textured icephobic studies, including the employed hydrophobic coatings and the reported adhesion strengths, are summarized in Table 1.

The literature, therefore, shows that improved anti-icing performance is commonly achieved by combining laser-generated hierarchical textures with low-surface-energy functionalization. However, direct comparison between the reported results remains difficult because surface morphology, substrate material, environmental conditions, icing protocols, and measurement methods vary substantially among studies. Moreover, most investigations evaluate only one selected coating–texture combination. Consequently, systematic comparisons of several coating chemistries applied to the same smooth and laser-textured substrate under identical testing conditions remain limited. Therefore, the present study addresses this knowledge gap by systematically evaluating how a bioinspired texture–chemistry design strategy performs on aluminum surfaces under two anti-icing-relevant metrics: freezing delay and ice adhesion strength. Substrates made of 1050A aluminum alloy are first laser-textured to produce a defined hierarchical surface topography. Subsequently, both bare and textured surfaces are functionalized using representative hydrophobic chemistries that include a fluoroalkyl phosphonic acid self-assembled monolayer and non-fluorinated alternatives based on alkyl phosphonic acids, fatty acids, and PDMS modification. Apparent static contact angles and roll-off angles are measured before and after icephobic testing to quantify not only water repellency but also the stability of the wetting state after icing. In addition, the surface free energy of coatings is evaluated on hydrophobized untextured samples. Icephobic performance is assessed using a unified protocol that combines freezing-delay measurements at −18 °C with ice adhesion measurements obtained from shear detachment of a 20 × 20 × 20 mm^3^ ice cube at −20 °C. Finally, SEM analysis is performed before and after testing to identify morphology changes and potential degradation features.

## 2. Materials and Methods

### 2.1. Surface Fabrication

Laser-textured and bare reference aluminum samples were prepared on aluminum plates (1050A H24, > 99.5% Al) with dimensions of 38 mm × 38 mm, and a thickness of 2 mm. For the core testing, 5 laser-textured and 5 untextured samples were prepared and evaluated in this study. Furthermore, to test the reproducibility and repeatability of the fabrication procedure and tests, an additional 10 samples were prepared, making a total of 22 samples tested in this study. One additional smooth specimen and one additional laser-textured specimen were prepared separately for surface roughness characterization, resulting in a total of 24 fabricated samples.

Firstly, bare reference samples were manufactured by the following procedure: they were first wiped with acetone and then thoroughly cleaned in isopropanol in an ultrasonic cleaner for 5 min, and they underwent a hydrophobization process using various hydrophobic coatings. Secondly, the other half of the aluminum plates were first laser-textured with a nanosecond fiber laser machine (JPT Opto-electronics Co., Ltd., Shenzhen, China, M7 30 W MOPA; λ  =  1064 nm) before undergoing hydrophobization. Nanosecond laser processing was selected because it enables multiscale roughness to be generated directly on aluminum in a controllable and reproducible manner, while allowing the same underlying morphology to be subsequently combined with different low-surface-energy coating chemistries. An F-Theta lens with a 70  ×  70 mm^2^ working area and a focal distance of 100 mm was used to focus the beam. The focused beam featured an approximate diameter of 25 μm on the surface during processing, with a manufacturer-reported laser beam quality of M^2^  <  1.3 and a maximum average laser source power of 30 W. Samples were prepared using equidistant parallel lines spaced 25 μm apart. The utilized power of the laser beam was 15 W, while the frequency was 110 kHz. The scanning velocity was 1600 mm s^−1^, the pulse width was 45 ns and the average pulse fluence was 23.9 J cm^−2^. After laser texturing, samples were cleaned in isopropanol in an ultrasonic cleaner for 10 min to remove the microparticles produced during the process that were not well adhered to the surface. Then, the surfaces were cleaned in the UV/ozone cleaner for 10 min to remove adsorbed volatile organic compounds. Finally, the fabricated samples underwent the hydrophobization process using different hydrophobic coatings. The hydrophobization process using various hydrophobic coatings on the surfaces is presented in Table 2.

### 2.2. Experimental Setup for Evaluation of Ice Adhesion Strength and Freezing Time Delay

**Figure 1:**
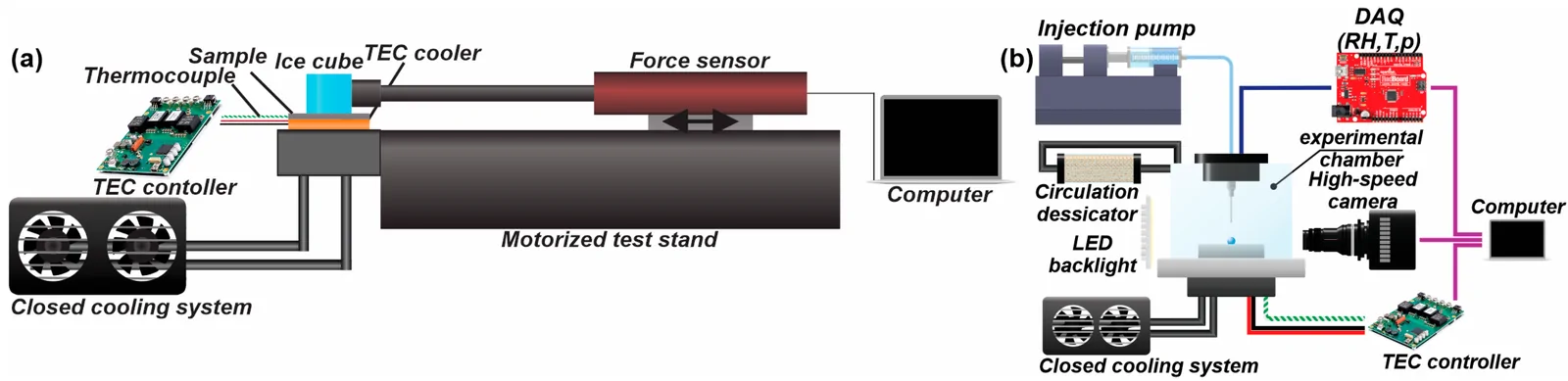
Custom-made experimental setup for evaluation of: (**a**) ice adhesion strength and (**b**) freezing time delay.

Ice adhesion measurements on the hydrophobized laser-textured and reference aluminum surfaces were conducted using the custom-made experimental setup shown in Figure 1. The experimental setup consisted of a motorized test stand (Axis STAH, Gdańsk, Poland) with a maximum force capacity of 500 N and a minimum linear displacement velocity of 10 mm min^−1^. A push–pull force sensor (Axis FC200) was mounted on the motorized test stand. Ice formation was achieved using a 40 × 40 mm^−2^ thermoelectric cooler (TEC) element (European Thermodynamics Ltd., Leicestershire, UK). The hot side of the TEC element was thermally stabilized using a Julabo FL1701 recirculating chiller (Seelbach, Germany). The sample temperature was monitored using a K-type thermocouple attached directly to the specimen surface, with a specified accuracy of ±0.2 °C. The measured temperature was used by the PID-controlled TEC system to regulate the specimen temperature. The TEC controller provided a temperature setpoint resolution of 0.01 °C, while both the prescribed setpoint and the measured specimen temperature were continuously monitored using the TEC control software. The plunger of the force sensor was equipped with a 3D-printed attachment to limit heat transfer from the ice cube and provide a large contact area close to the ice-cube base. This configuration limited the formation of secondary tensile and compressive stress fields beneath the ice cube and ensured that shear was the predominant loading mode. The distance between the bottom of the plunger attachment and the surface was less than 1 mm.

### 2.3. Evaluation of Ice Adhesion Strength and Freezing Time Delay

The ice cube on top of the hydrophobized reference and laser-textured surfaces was formed using a silicone mold with inner dimensions of 20 × 20 × 20 mm^3^. Distilled water supplied by Carl ROTH (Karlsruhe, Germany), with a conductivity of ≤2.0 μS cm^−1^ and a pH value of 5.0–7.0, was used for the preparation of the water droplets and ice cubes in all anti-icing measurements. The silicone mold was filled with distilled water at room temperature, and then the TEC cooler was utilized to cool down the surface to −20 °C and freeze the water within the silicone mold to form the ice cube. The ice adhesion measurements were performed at an ambient temperature of 23–26 °C and a relative humidity of 40–60%. The water was added to the silicone mold before cooling was initiated to prevent frost formation on the specimen surface prior to freezing. The mold was covered during freezing to reduce the influence of ambient airflow and other external disturbances. After approximately 20 min, when the water had completely solidified, the cover and silicone mold were removed, and the motorized test stand bearing the force sensor was moved towards the ice cube at $10\;mm\;min^{-1}$. The maximum force achieved before the ice cube was sheared off the surface was noted and used to calculate the shear strength (i.e., ice adhesion). For each coating–morphology condition, measurements were performed on two physically separate specimens. Ten consecutive icing–detachment cycles were performed on each specimen, resulting in 20 ice adhesion strength measurements per condition. The two samples represent independent physical sample replicates, whereas the ten consecutive measurements performed on each specimen represent repeated measurements of the same experimental unit. Ice adhesion strength was calculated as

(1) $$\tau_{ice}=\frac{F_{max}}{A},$$

where *F*_max_ is the maximum force recorded immediately before ice detachment, and *A* is the nominal ice–surface contact area. The contact area was defined by the internal dimensions of the silicone mold, 20 × 20 mm^2^, corresponding to *A* = 400 mm^2^. The force measurements were performed using a digital force sensor with a resolution of 0.05 N and a specified accuracy of ±0.2 N. For the nominal ice–surface contact area of 400 mm^2^, these values correspond to an ice adhesion strength resolution of 0.125 kPa and a force-sensor accuracy contribution of ±0.50 kPa. Relative to the lowest measured detachment force of 9.05 N, the relative force resolution and accuracy were 0.55% and 2.21%, respectively. The force-sensor accuracy was considered separately from the experimental variability between specimens and repeated icing–detachment cycles, which are reported as the standard deviation of the 20 measurements obtained for each condition.

The freezing delay of water droplets on the fabricated surfaces was evaluated by placing sixteen droplets (*V* = 7 μL) on each surface at room temperature. The surface was then cooled at a nominal rate of 1 K s^−1^ with successive temperature setpoints of 5 °C, 0 °C, and −18 °C. The freezing delay was measured from the moment cooling was initiated. Droplets that froze before the final target temperature was reached were also included in the analysis. The target temperature of −18 °C was selected as a representative condition within the range of −15 to −20 °C previously used for freezing-delay measurements employing a comparable controlled-cooling protocol [9,40]. The freezing-delay measurements were performed at an ambient temperature of 23–26 °C, while the relative humidity inside the closed test chamber was maintained below 10% to minimize condensation and frost formation during cooling. Freezing-delay measurements were performed on two physically separate samples for each coating–morphology condition. Sixteen droplets were simultaneously monitored on each sample during one cooling run, resulting in 32 droplet observations per condition.

A different temperature condition was used for the ice adhesion measurements because this test required the complete and reproducible solidification of a macroscopic ice cube before mechanical detachment. Therefore, the TEC setpoint was changed to −20 °C, and the water was allowed to freeze for approximately 20 min before the mold was removed and the shear-detachment test was initiated. Thus, the temperatures used in the two experiments reflect the different requirements of the freezing-delay and ice adhesion protocols.

### 2.4. Surface Analysis

The evaluation of surface wettability of functionalized samples was performed by measuring apparent static contact angles and roll-off angles using distilled water droplets with a volume of 8 µL before and after the ice adhesion test. The evaluations of apparent static contact angles and roll-off angles were made using a custom-made experimental setup at room temperature. During measurements of apparent static contact angle, five water droplets were placed on different spots on each surface, and the captured droplet images were analyzed using Ossila Contact Angle software (version v3.1.2.2). During the measurements of the roll-off angle, five droplets on different spots on the surface were rolled off and the angle was recorded. Prior to chemical functionalization, the laser-textured aluminum surfaces were superhydrophilic, with an apparent static water contact angle below 5°, and the deposited water droplets spread almost immediately across the surface. The measurements of contact angles before and after ice adhesion strength tests are shown in Table 3. In Table 3, average values of 10 measurements for each sample are shown, with five measurements performed on each of the two identical samples to ensure the reproducibility and repeatability of the manufactured surfaces.

From Table 3, it can be observed that the hydrophobized laser-textured (LT) surfaces functionalized with different hydrophobic coatings exhibit apparent static water contact angles above 150°. However, not all LT surfaces demonstrated roll-off angles below 10° prior to the ice adhesion strength tests. In contrast, the reference (REF) surfaces coated with the same hydrophobic agents showed apparent static contact angles in the range of 97–125°, with roll-off angles well above 10°, indicating strong droplet pinning and a high-adhesion wetting state. The representative image of static contact angles is shown in Figure S1 in Section S1 of the Supplementary Materials.

After the ice adhesion tests, the contact-angle measurements indicate that most functionalized surfaces underwent degradation. In particular, although the LT surfaces functionalized with FDPA and HDPA retained apparent static contact angles above 150°, their roll-off angles increased markedly (>10°), meaning that the surfaces no longer maintained a superhydrophobic low-adhesion state. This trend suggests a shift from a Cassie–Baxter state toward a mixed or Wenzel wetting regime after icing, i.e., partial/complete liquid impalement of the laser-generated texture. Such transitions increase contact-angle hysteresis and droplet pinning, so high apparent static contact angles may persist even when roll-off angles rise substantially. For coatings that fell below 150° after testing, the results indicate a more pronounced loss of the Cassie state and/or degradation of the hydrophobic layer. Likewise, the reduced contact angles on the coated reference surfaces are consistent with coating degradation, while their persistently high roll-off angles indicate a predominantly high-hysteresis wetting state throughout.

**Figure 2:**
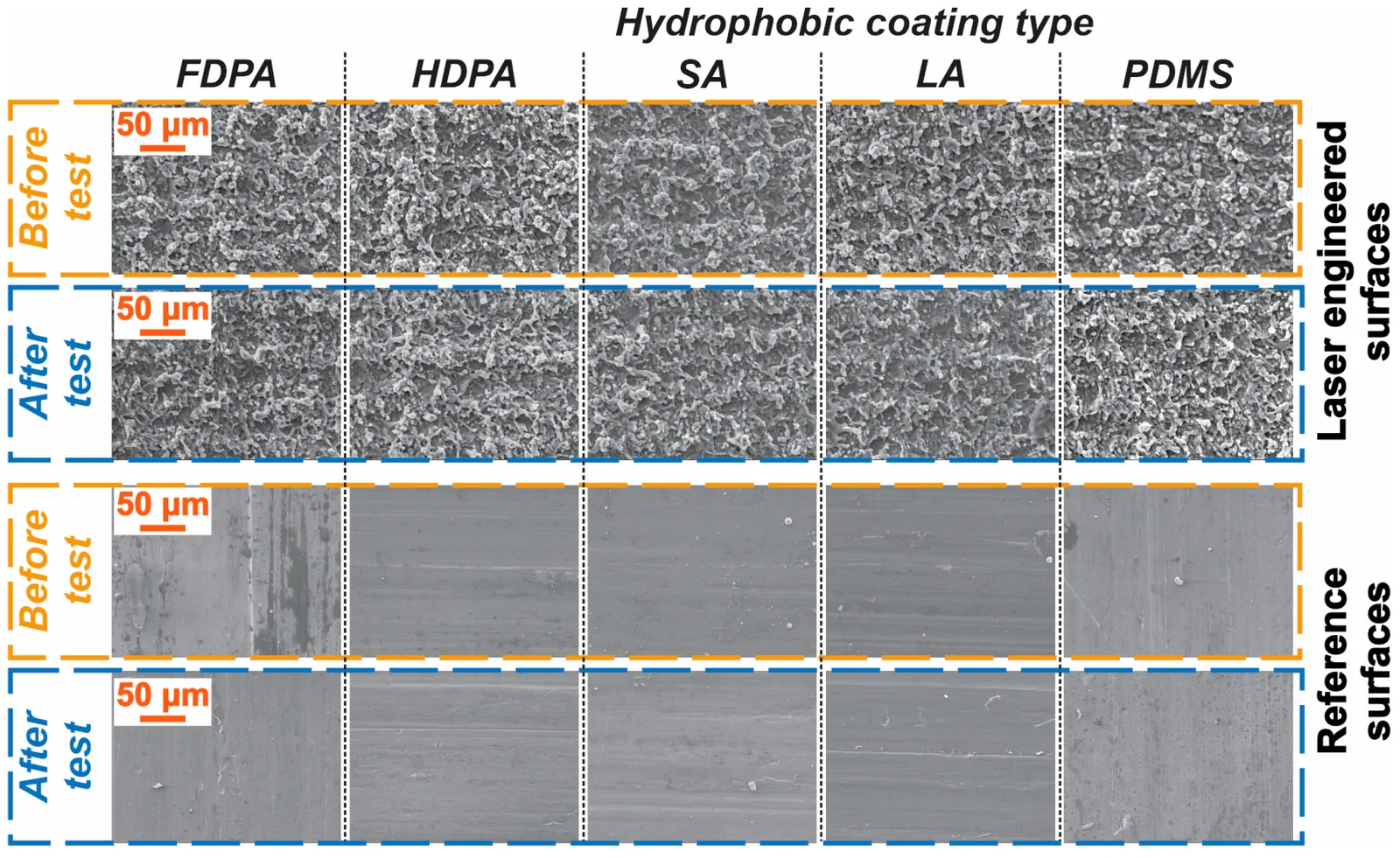
SEM images of laser-textured and reference surfaces functionalized utilizing different hydrophobic coatings before and after ice adhesion tests.

Furthermore, the analysis of the surface morphology and topography before and after the ice adhesion tests was performed using scanning electron microscopy (SEM). The SEM images of the tested surface are shown in Figure 2. Representative cross-sectional SEM images and a three-dimensional optical topography map are provided in Figure S2 in Section S1 of the Supplementary Materials to further illustrate the morphology of the reference and laser-textured surfaces. One can observe that a huge difference in surface topography and morphology before and after was not observed; therefore, the main difference between the tested surfaces is relies on the different hydrophobic monolayers applied using different hydrophobic chemicals, ranging from from fluorinated to fully green.

Surface roughness of the reference and laser-textured samples prior to the anti-icing measurements was characterized using a Keyence VHX-6000 optical microscope (Mechelen, Belgium). The measurements were performed on separate samples from those used in the anti-icing tests, since the applied monolayer coating is not expected to alter the surface roughness. Therefore, one reference sample and one laser-textured sample were prepared for roughness characterization.

The line roughness parameters were measured at a magnification of 200×. At each selected location, twenty line roughness profiles were acquired at two distinct locations, giving a total of 40 measurements per sample. For the reference surface, the measured profile roughness values were *R*_a_ = 1.10 ± 0.12 μm and *R*_z_ = 6.60 ± 1.99 μm, where the reported variation reflects spatial measurements performed on the same sample. To evaluate sample-to-sample reproducibility of the laser-texturing process, roughness measurements were additionally performed on three independently fabricated laser-textured specimens. The resulting specimen-averaged values were *R*_a_ = 5.71 ± 0.20 μm and *R*_z_ = 25.71 ± 2.15 μm, where the standard deviations represent between-specimen variation.

The corresponding areal roughness values for the reference surface were *S*_a_ = 1.62 ± 0.20 μm and *S*_z_ = 17.34 ± 4.40 μm. Across the three independently fabricated laser-textured samples, the areal roughness values were *S*_a_ = 5.91 ± 0.20 μm and *S*_z_ = 34.91 ± 6.75 μm. The larger variation observed for *S*_z_ is attributed to the sensitivity of this extreme-height parameter to isolated peaks and valleys within the irregular laser-generated morphology.

### 2.5. Surface Free Energy Measurements

**Figure 3:**
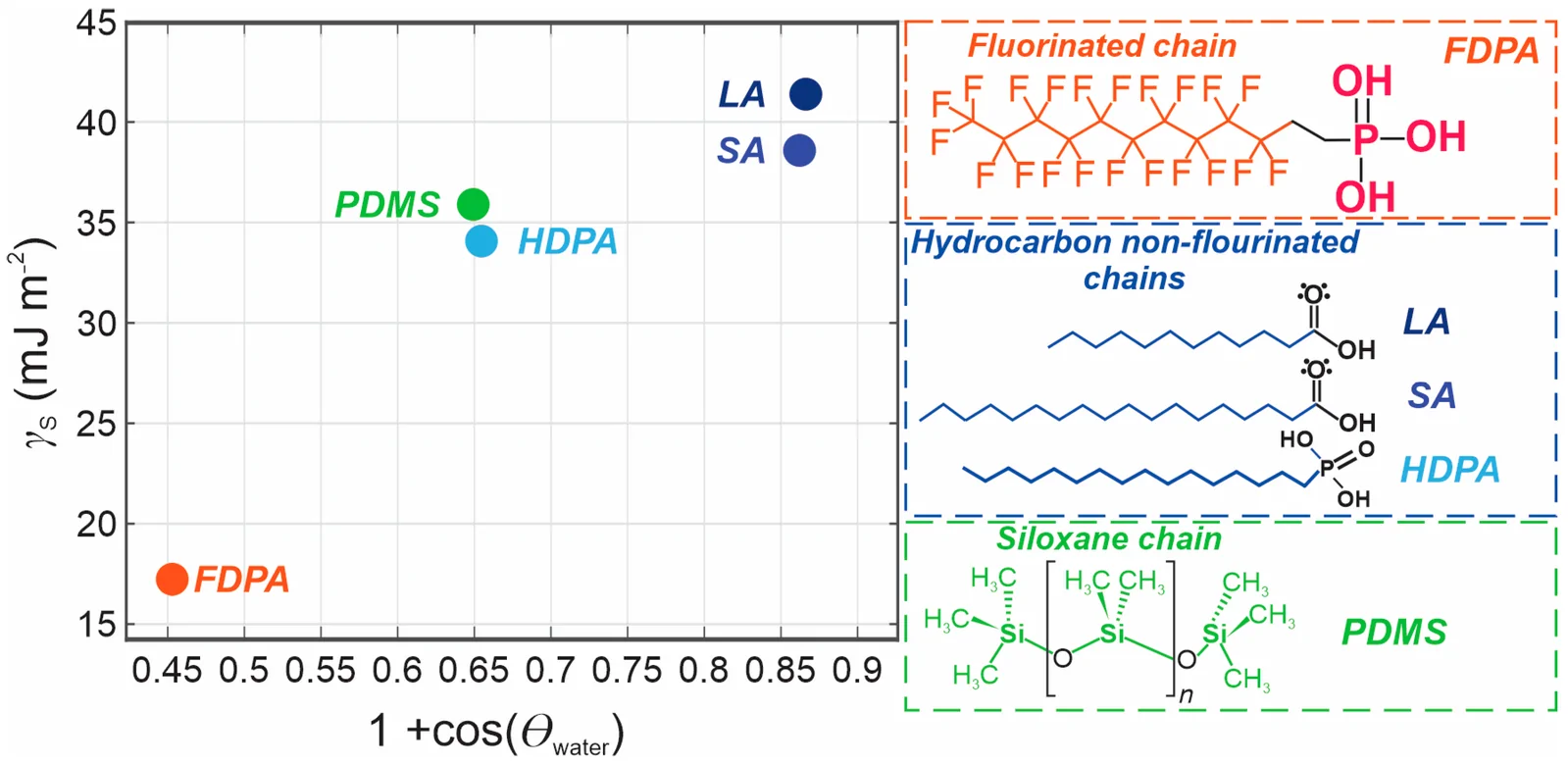
The surface free energy of the investigated hydrophobic monolayer coatings.

The surface free energy of the hydrophobic monolayer coatings on reference aluminum surfaces was estimated using static contact-angle measurements with two different liquids, i.e., distilled water and diiodomethane, and the Owens–Wendt–Rabel–Kaelble (OWRK) method. For the calculation of the surface free energy, static contact angle measurements were performed using distilled water and diiodomethane. On each surface, 10 measurements were carried out, and the average contact angle values were used for the surface free energy calculation. The measurements of static contact angle for water are reported in Table 3, while the measurements of static contact angle for diiodomethane are reported in Table S1 in the Supplementary Materials, Section S2. The dispersive and polar components of the surface free energy for each tested reference surface are listed in Table S2 in the Supplementary Materials, Section S2. Utilizing this approach, the solid surface free energy is decomposed into dispersive and polar contributions:

(2) $$\gamma_{S}=\gamma_{S}^{D}+\gamma_{S}^{P},$$

and related to the measured apparent static contact angle *θ* of the used liquids through

(3) $$\gamma_{L}\left(1+\cos\theta \right)=2\left(\sqrt{\gamma_{S}^{D}\gamma_{L}^{D}}+\sqrt{\gamma_{S}^{P}\gamma_{L}^{P}}\right),$$

where $\gamma_{L}$ is the total surface tension of the test liquid and $\gamma_{L}^{D}$ and $\gamma_{L}^{P}$ are its dispersive and polar components. Diiodomethane is essentially apolar; its contact angle provides a direct estimate of $\gamma_{S}^{D}$, after which $\gamma_{S}^{P}$ is obtained from the water measurement, yielding the total $\gamma_{S}$. The surface free energy of the investigated hydrophobic monolayer coatings formed on the aluminum surface, evaluated over the corresponding measured water contact angles, is presented in Figure 3.

As shown in Figure 3, the FDPA monolayer exhibits the lowest surface free energy. This trend is consistent with the established understanding that fluorinated terminal groups form very low-energy interfaces because the outermost fluorocarbon groups interact only weakly with other materials compared with hydrocarbon groups [41]. In contrast, the HDPA and PDMS monolayers are non-fluorinated, and their outermost chemical groups therefore provide stronger dispersive interactions than fluorinated groups. Consequently, the measured surface free energy increases compared with FDPA [42]. The lauric acid and stearic acid monolayers show the highest surface free energy among the tested coatings. A practical reason is that fatty acid monolayers on oxidized aluminum can be more sensitive to processing and may form films with a higher density of defects or incomplete coverage than phosphonic acid-based monolayers, which increases the effective contribution of the underlying polar aluminum oxide and leads to a higher apparent surface free energy obtained from contact-angle-based models [43]. Finally, the surface free energy for stearic acid is slightly lower than that for lauric acid, which is consistent with the longer hydrocarbon chain providing more effective screening of the polar interface and promoting a more hydrophobic outer surface compared with the shorter-chain fatty acid.

## 3. Results

### 3.1. Freezing Delay Performance

**Figure 4:**
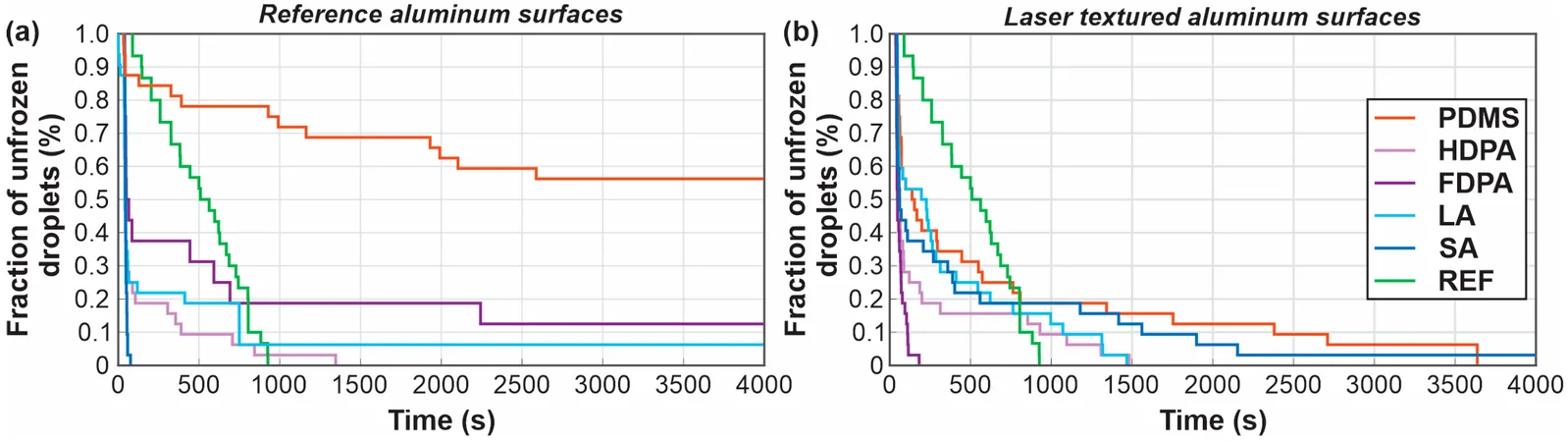
Freezing time delay of (**a**) functionalized reference surfaces and (**b**) functionalized laser-textured surfaces.

The typical metric used to evaluate the anti-icing performance of functionalized surfaces is the temporal freezing-delay. In this study, the freezing delay of functionalized bare and laser-textured aluminum surfaces was evaluated by monitoring the time required for complete droplet freezing at −18 °C. For each surface, sixteen water droplets were placed on the sample, and the freezing time of each droplet was recorded. To assess repeatability, the same procedure was repeated on a second nominally identical specimen for each condition, resulting in a total of 32 droplets per surface. If a droplet did not freeze within the one-hour observation window, the experiment was terminated and the corresponding observation was treated as right-censored at 3600 s. The freezing delay performance of the bare reference aluminum surfaces is shown in Figure 4a. Accordingly, the curves should be interpreted not only through the mean freezing time but also through the early time decay and the terminal survival level, which together reflect both the probability of early nucleation events and the persistence of long-lived unfrozen droplets. Furthermore, it is important to note that all reference surfaces had identical topography and roughness; therefore, the differences observed in freezing time delay on these surfaces may be attributed primarily to the hydrophobic layer chemistry and the resulting interfacial nucleation conditions, rather than to variations in surface roughness or topography.

For the non-functionalized reference aluminum surface, the mean freezing-delay time obtained from 32 measurements was 515 s, and approximately 47% of the droplets froze before this mean value. The reference surfaces functionalized with fatty acids exhibited shorter freezing delays than the uncoated surface. The mean freezing delay was 148 s for REF-LA and 48 s for REF-SA, with approximately 69% and 78% of droplets freezing before their corresponding mean values, respectively. The phosphonic acid functionalized reference surfaces showed intermediate freezing delays, with mean values of 208 s for REF-FDPA and 163 s for REF-HDPA. For these surfaces, approximately 69% of droplets froze before the mean for REF-FDPA, while 81% froze before the mean for REF-HDPA. In contrast, the PDMS modified reference surface showed the longest freezing delays, with a mean value of 907 s, and only 22% of droplets froze before this mean. Notably, a large fraction of droplets on REF-PDMS remained liquid at the end of the one-hour test, as indicated by the high terminal level in Figure 4a. A complementary Kaplan–Meier survival analysis accounting for droplets that remained unfrozen after 3600 s, including median freezing times, restricted mean survival times, and log-rank comparisons, is provided in Table S3 in Section S3 of the Supplementary Materials.

The observed differences can be explained by considering heterogeneous ice nucleation at the solid–liquid interface. On smooth metals, freezing initiation is typically governed by a limited number of active nucleation sites such as exposed polar oxide regions, defects, contaminants, and chemically heterogeneous domains. As a result, macroscopic wettability alone is not sufficient to predict freezing delay, because nucleation is governed by a limited number of localized heterogeneous nucleation sites at the interface, such as defects, exposed polar regions, impurities, or coating discontinuities, rather than by the apparent contact angle. The surface free energy analysis further supports this interpretation. The calculated surface free energy components indicate that the coatings differ not only in total surface free energy but also in the polar contribution. A lower polar component implies weaker specific interactions between water and the surface and a reduced tendency to form strongly bound interfacial water structures that can stabilize an incipient ice embryo. Therefore, coatings with lower polar surface energy are expected to suppress heterogeneous nucleation and to increase freezing delay, which is consistent with the long-time tail observed for REF-PDMS. In this context, the elevated terminal survival level for REF-PDMS indicates a reduced freezing hazard throughout the observation window rather than merely a shift in the average freezing time. In contrast, thin organic layers formed by fatty acids and phosphonic acids can contain local defects, incomplete coverage, or interfacial chemical heterogeneity. Even if the total surface free energy is reduced compared with bare aluminum, locally exposed polar sites and domain boundaries can still act as efficient nucleation triggers. Because the deposition routes and post-treatment steps differ among coatings, variations in monolayer packing density and interfacial uniformity may contribute to the distribution of potential nucleation sites and thereby influence the freezing delay. Overall, the reference surface results indicate that coating chemistry controls freezing delay primarily through its influence on the density and effectiveness of nucleation sites and through the polar component of the surface free energy, rather than through wettability alone.

The freezing delay performance obtained on the laser-textured aluminum surfaces is shown in Figure 4b. In comparison with the smooth reference substrates, the curves shift toward shorter times and exhibit a steeper initial decrease, which indicates that laser texturing increases the probability of early freezing events. This behavior is consistent with the fact that the laser-generated topography increases the real surface area and introduces a higher density of potential heterogeneous nucleation regions, including asperity edges, microcavities, and locally heterogeneous regions created during texturing and subsequent functionalization. Although the entire textured area is not necessarily in direct contact with the droplet under an initial Cassie–Baxter wetting state, local liquid penetration during cooling can increase the effective solid–liquid contact and activate such regions as nucleation-promoting sites. In addition, under subzero conditions, the texture can promote local condensation and capillary imbibition of water into the surface features, which increases the solid–liquid contact fraction and reduces the effectiveness of the hydrophobic chemistry in delaying nucleation. This is particularly important for laser-textured surfaces because the apparent superhydrophobic state measured at room temperature does not necessarily remain stable during cooling. Condensed water or frost formed inside the micro/nanostructure can replace the initially trapped air pockets, increase the effective ice–solid contact area, and generate additional mechanical anchoring after freezing [10,31,44,45,46]. Therefore, although the LT surfaces initially exhibit high apparent contact angles, their freezing-delay performance can become poorer than that of the corresponding REF surfaces because the textured morphology provides additional sites for condensation, frost nucleation, and subsequent ice locking within the surface features. Therefore, the anti-icing performance on the laser-textured surfaces is controlled not only by coating chemistry but also by the wetting state within the texture. Similar to the functionalized reference surfaces, laser-textured surfaces had identical morphology; the differences among coatings reflect how each monolayer chemistry modifies the nucleation probability under textured wetting conditions.

The poorest freezing delay performance on the laser-textured surfaces was observed for FDPA. All droplets froze within a short time window, and the unfrozen fraction rapidly approached zero, which is reflected by the sharp drop in the survival curve. This suggests that the fluorinated phosphonic acid monolayer did not prevent the activation of nucleation sites on the textured surface. A plausible explanation is that, on a highly structured substrate, the coating chemistry influences not only the apparent room-temperature wettability but also the condensation and frosting behavior within the texture under subzero conditions. In particular, on surfaces with very low surface free energy, such as LT-FDPA, water vapor may more readily condense as discrete droplets or frost within the microstructured topography. Once such condensed water penetrates or bridges the texture, it can promote freezing and thereby shorten the freezing delay. From this perspective, the poor freezing delay performance of LT-FDPA may reflect condensation- and frost-mediated wetting of the texture rather than the intrinsic hydrophobicity of the coating itself. The HDPA-modified laser-textured surface showed a slightly improved response relative to FDPA but still exhibited a pronounced early-time decay of the unfrozen fraction, suggesting that similar mechanisms remained relevant, although less pronounced.

The fatty acid functionalized laser-textured surfaces, namely LA and SA, showed intermediate freezing-delay behavior. The survival curves decreased rapidly during the initial period and then continued to decay more gradually, which is consistent with a mixed response where some droplets freeze early due to local penetration of the texture or exposure of polar sites, while other droplets experience delayed nucleation. This behavior is compatible with thin organic acid layers that can be sensitive to coverage heterogeneity on complex topographies, particularly within recessed regions of the texture. In contrast, the PDMS modified laser-textured surface exhibited the longest freezing delays among the coated textured samples and retained the highest unfrozen fraction at long times, although the tail was less pronounced than on the smooth PDMS coated reference surface. This trend may be attributed to the formation of a CVD-derived PDMS monolayer that presents a siloxane-terminated, low-polarity interface. Such a surface chemistry may reduce specific water–surface interactions and, consequently, decrease the probability of activating efficient heterogeneous nucleation sites, which is consistent with the higher unfrozen fraction observed for PDMS at long times. However, the persistence of freezing events over the full observation window indicates that texture-driven effects, such as partial wetting of microfeatures and enhanced pinning within the topography, still promote nucleation compared with smooth substrates.

Overall, the results observed on laser-textured surfaces demonstrate that reducing surface free energy and increasing static contact angle are not sufficient to guarantee long freezing delays when the surface contains micro and nanostructures. Instead, the dominant factor becomes whether the texture remains in a low solid fraction wetting state during cooling or transitions toward a wetted state that exposes high-energy nucleation sites. This also explains why the differences among coating chemistries become less pronounced on the textured surfaces than on the smooth reference surfaces, and why PDMS remains the most effective coating for freezing delay, even though its benefit is partly diminished by texture-induced wetting and nucleation pathways. In the context of bioinspired superhydrophobicity, this result shows that hierarchical roughness alone is not a sufficient anti-icing design criterion. The biologically inspired water-shedding function is beneficial only if the texture preserves a low solid–liquid contact fraction during cooling and does not become a site for condensation-, frost-, or penetration-mediated nucleation.

### 3.2. Hydrophobic Coating Influence on Ice Adhesion Strength

**Figure 5:**
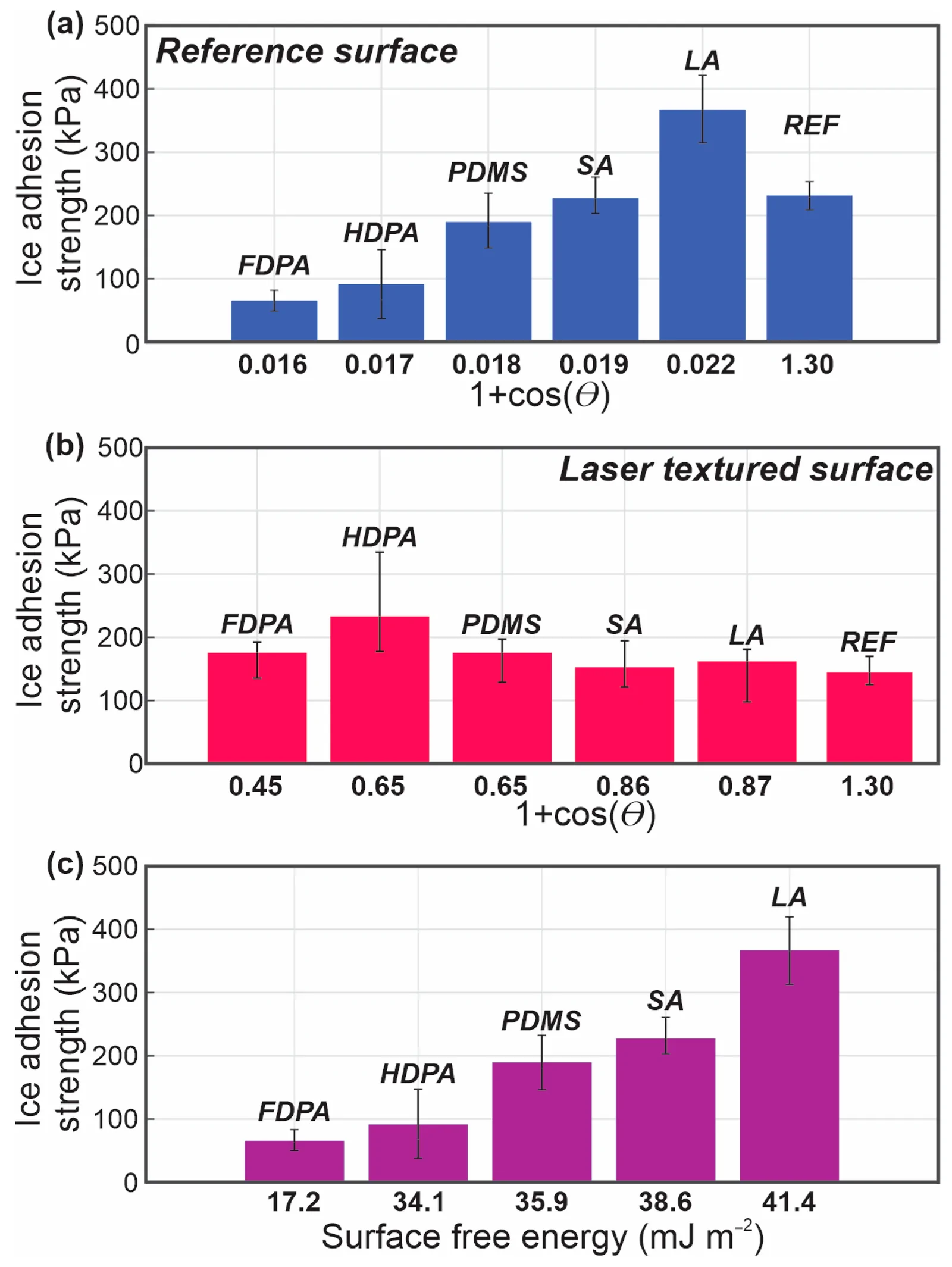
The ice adhesion strength on (**a**) reference and (**b**) laser-textured aluminum surfaces functionalized using different monolayer coatings. (**c**) The dependence of ice adhesion strength on the surface free energy of the functionalized reference surfaces.

The ice adhesion strength, measured for the functionalized reference aluminum surfaces and the functionalized laser-textured aluminum surfaces, is summarized in Figure 5a and Figure 5b, respectively. The corresponding statistical scatter is represented using quartile values. The dependence of ice adhesion strength on the surface free energy of the functionalized reference surfaces is shown in Figure 5c.

For the reference surfaces, all samples have identical surface morphology and roughness. Therefore, differences in ice adhesion strength primarily reflect the influence of the hydrophobic layer chemistry and the resulting interfacial surface free energy, rather than morphology-driven mechanical effects. The largest reduction in ice adhesion strength was obtained for the surface functionalized with fluorinated phosphonic acid (FDPA), where the ice adhesion strength decreased by approximately 72% relative to the non-functionalized reference aluminum. A pronounced reduction was also achieved for the surface functionalized with the non-fluorinated phosphonic acid (HDPA), with a decrease of approximately 50% relative to the reference. In contrast, the PDMS modification provided only a limited reduction in ice adhesion strength, while functionalization with fatty acids did not yield a consistent improvement. In particular, the surface functionalized with stearic acid remained close to the non-functionalized reference level, whereas the surface coated with lauric acid resulted in higher ice adhesion strength than the reference surface. Therefore, these observations show that lowering ice adhesion strength on smooth aluminum is not guaranteed by increasing static hydrophobicity alone. Instead, the outcome depends on the detailed interfacial chemistry and on the degree to which the coating forms a uniform and low-polarity interface toward water and ice.

The surface free energy representation in Figure 5c supports this interpretation. The lowest ice adhesion strength coincides with the lowest surface free energy coating (FDPA), whereas coatings with higher surface free energy generally exhibit higher ice adhesion strength. This trend is consistent with reports showing that ice adhesion strength on smooth rigid samples correlates with the practical work of adhesion for water and that dynamic wetting parameters are often more relevant than static contact angles when describing adhesion and detachment behavior. In this context, the stronger response of FDPA compared with HDPA is consistent with the additional reduction of interfacial interactions obtained by fluorinated terminal groups, which typically lower surface free energy and reduce the contribution of polar bonding at the ice–solid interface [47].

The weak and inconsistent response of fatty acid functionalization can be explained by differences in how these layers bind and organize on aluminum oxide. Phosphonic acids are widely reported to form robust and well-defined monolayers on aluminum and aluminum oxide surfaces, while carboxylic acids generally exhibit weaker binding and are more sensitive to partial coverage, exchange reactions, and local non-uniformity [48,49,50]. When ice adhesion is controlled by local heterogeneity, a limited number of exposed polar sites, domain boundaries, or defect-rich regions can dominate the measured response even if the surface appears hydrophobic in static measurements [47]. Therefore, the higher ice adhesion strength obtained for LA compared with SA is consistent with the expectation that shorter-chain organic layers tend to exhibit a higher probability of locally exposed sites and lower packing quality than longer-chain analogues, which increases the likelihood of interfacial pinning during shear detachment [49].

The ice adhesion results obtained on the laser-textured surfaces are shown in Figure 5b. In comparison with the reference surfaces, the relative differences among coating chemistries are less pronounced, and the overall reductions relative to the non-functionalized reference surface are substantially smaller. For the textured substrate, SA and HDPA provide the most favorable response, corresponding to roughly a 40% reduction relative to the non-functionalized reference surface, while PDMS and FDPA yield intermediate reductions of approximately 30%. In contrast, LA provides only a minor reduction relative to the reference surface and remains the least effective chemistry among the set of laser-textured surfaces. Importantly, the trend observed on the textured surfaces differs from the trend on the smooth reference surfaces, and this trend does not follow a simple monotonic dependence on static wettability. This indicates that, once micro- and nanotopography is introduced, ice adhesion strength is no longer governed solely by surface energy or apparent contact angle. The distinct mechanisms underlying this decoupling, including heterogeneous nucleation, liquid penetration, interfacial crack initiation, and mechanical interlocking, are schematically summarized in Figure S3, Section S4 in the Supplementary Materials.

This behavior is consistent with studies showing that superhydrophobic wetting observed at room temperature does not necessarily lead to low ice adhesion when freezing occurs under conditions that promote wetting of the texture. Chen et al. demonstrated that even for superhydrophobic surfaces, ice adhesion can remain high because the relevant interface during freezing is governed by the actual ice–solid contact fraction and the interfacial failure path during shear removal rather than by static contact angle values [51]. Under the present conditions, the laser-generated microcavities and asperity edges can promote local penetration of water prior to freezing. Once freezing occurs inside recessed regions, the effective ice–solid contact increases and the contribution of mechanical engagement becomes significant. Therefore, differences in coating chemistry become less dominant, and the ice adhesion strength values converge, as observed in Figure 5b.

In addition, frost formation and condensation within surface textures can compromise icephobic behavior because ice formed inside the texture increases the effective contact area and enhances mechanical engagement [45]. This mechanism provides a consistent explanation for the narrower range of ice adhesion values observed among the textured samples, even though the same coatings produce pronounced differences on the smooth reference substrates. At the same time, the fact that SA and HDPA provide the most favorable response on the textured substrate suggests that, at least for the present surface morphology, the governing factor is not only intrinsic surface free energy but also how each chemistry influences wetting stability within the texture during cooling and freezing.

The wetting characterization before and after the ice adhesion tests (see Table 3) provides additional evidence that the wetting state of the textured surfaces is not fully preserved during icing and shear removal. Prior to testing, all laser-textured coatings exhibited superhydrophobic apparent contact angles and low roll-off angles, indicating a low-hysteresis state. After the adhesion tests, several textured samples showed a pronounced increase in roll-off angle, in some cases to values above 90°, together with a decrease in apparent contact angle. This change is most evident for LT-SA and LT-LA, where the post-test roll-off angle exceeded 90° and the apparent contact angle decreased markedly, which indicates a transition toward a pinned, high-hysteresis state. Such behavior is consistent with partial loss of the Cassie-type wetting state due to liquid penetration and freezing within the texture, local damage, or removal of the functional layer from asperity peaks during the detachment event. A similar loss of dynamic water repellency after repeated icing or de-icing has been reported for laser-fabricated textures when local wetting of microfeatures occurs [9]. In contrast, LT-FDPA retained a very low roll-off angle after testing, with only a minor change in apparent contact angle, which suggests that this chemistry better preserves the low-hysteresis wetting state on the present texture. The remaining coatings show intermediate behavior, where occasional post-test roll-off angles below 90° were observed alongside pinned droplets, which indicates spatial variability of the wetting state in the tested area. Overall, the roll-off measurements confirm that the ice adhesion test is a severe perturbation for textured superhydrophobic surfaces and that preserving a low-hysteresis state during icing is a key requirement for sustaining low ice adhesion strength.

Furthermore, a two-way analysis of variance (ANOVA) was performed to evaluate the effects of surface topography, coating chemistry and their interaction on ice adhesion strength. The analysis showed a statistically significant effect of coating chemistry (*p* = 9.41 × 10^−21^) and a statistically significant morphology–chemistry interaction (*p* = 4.32 × 10^−8^), whereas the main effect of surface topography was not statistically significant when averaged across all coatings (*p* = 0.108). Therefore, the influence of surface roughness on ice adhesion strength is coating-dependent, which is consistent with the coating-specific changes observed when comparing each reference surface with its laser-textured counterpart, as discussed in the following section.

Overall, the present results indicate that the contribution of hydrophobic functionalization is fundamentally different for the reference and laser-textured surfaces. On the smooth reference surfaces, where the morphology is unchanged, the measured differences in ice adhesion strength are governed primarily by the chemistry of the hydrophobic layer and the resulting interfacial surface free energy. In contrast, for the laser-textured surfaces, the hydrophobic treatment still provides an additional reduction in ice adhesion strength, but the dominant role is played by the surface texture itself. In other words, once micro- and nano-structured roughness is introduced, the final ice adhesion response is controlled mainly by texture-mediated wetting, liquid penetration, and mechanical interlocking during freezing and detachment, while the applied coating acts as a secondary modifying factor. Lastly, the ice adhesion strength values on superhydrophobic laser-textured surfaces converged to roughly the same value, confirming that the surface morphology and not the monolayer coating represent a limiting factor (i.e., the lowest achievable ice adhesion strength on a surface with significant roughness).

The combined freezing delay and ice adhesion results show that these two metrics probe different controlling mechanisms and therefore do not necessarily rank coatings in the same order. Freezing delay is governed by the probability of initiating heterogeneous nucleation at a limited number of active sites, whereas ice adhesion strength is governed by interfacial fracture after ice has fully formed. This distinction explains why PDMS exhibits the most favorable freezing delay response, yet does not provide the lowest ice adhesion strength on the reference surface. On textured substrates, this decoupling becomes more pronounced because wetting and freezing within microfeatures can dominate adhesion regardless of nucleation behavior. This decoupling is particularly important for biomimetic anti-icing design because natural superhydrophobicity is primarily a liquid-repellency strategy, whereas engineering icephobicity also requires control over the frozen interface and the mechanical failure path during detachment.

### 3.3. Surface Roughness Influence on Ice Adhesion Utilizing Various Hydrophobic Coatings

**Figure 6:**
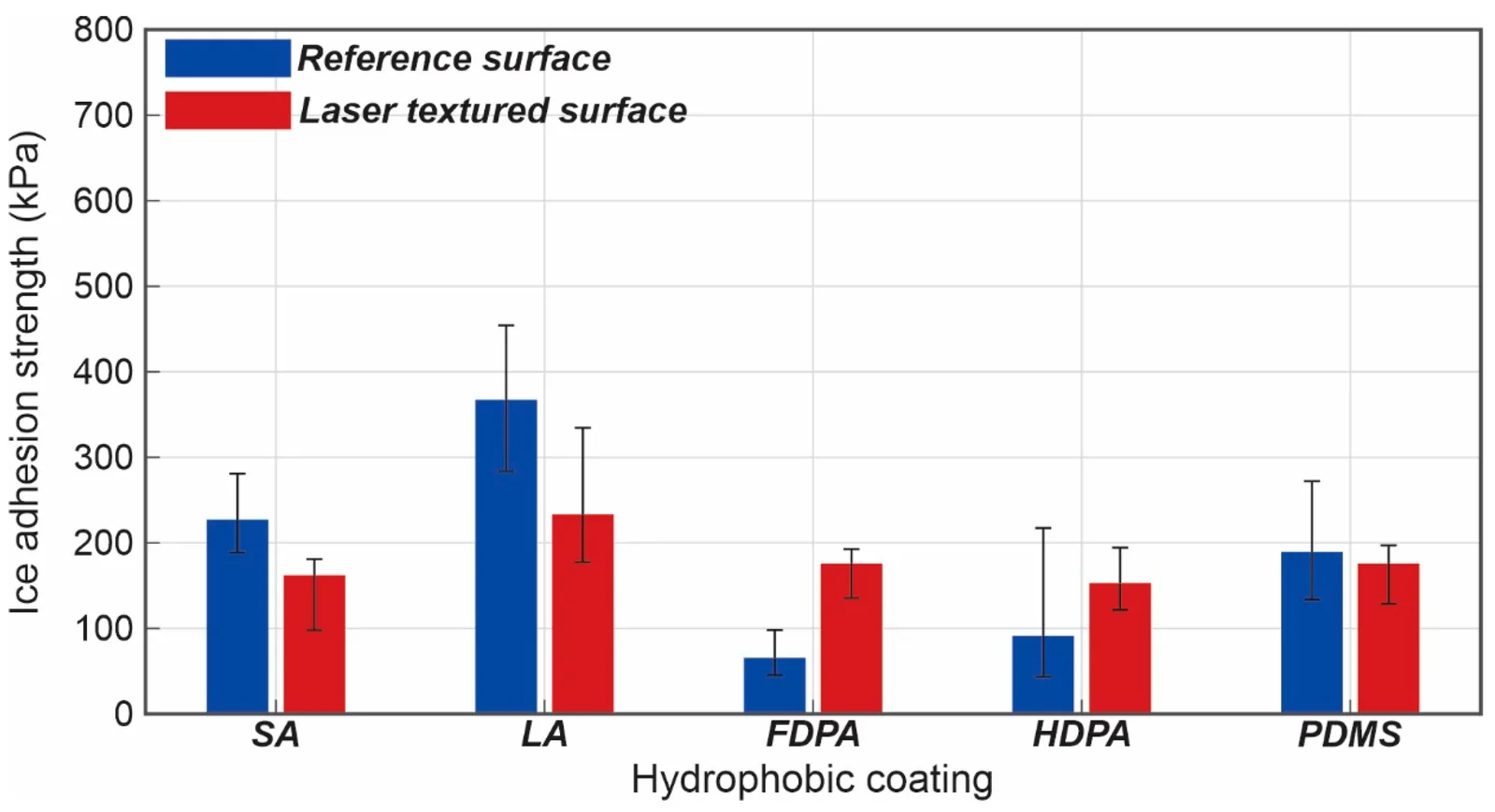
Influence of surface roughness on ice adhesion for different hydrophobic coatings.

The influence of surface roughness on ice adhesion strength was evaluated by comparing pairs of samples with identical surface chemistry but different morphology, namely the smooth reference surface and the laser-textured surface. Since each pair was functionalized using the same hydrophobic chemistry, the difference between the smooth and textured responses isolates the effect of roughness on ice adhesion strength for a given coating. The comparison of ice adhesion strength obtained on reference and laser-textured surfaces for different coatings is shown in Figure 6.

The results show that the effect of increased roughness is coating-dependent and not monotonic. For FDPA and HDPA, the laser-textured surfaces exhibit higher ice adhesion strength than the corresponding smooth reference surfaces. This indicates that, for phosphonic acid functionalization, introducing laser-generated topography adds an adhesion contribution that outweighs the chemical benefit observed on the smooth substrate. In contrast, for SA and LA, laser texturing reduces ice adhesion strength relative to the smooth reference surfaces, with the most pronounced reduction observed for LA. For PDMS, the difference between the smooth and textured surfaces is comparatively small, which suggests that the dominant contribution to detachment remains similar on both morphologies for this hydrophobic coating.

These trends indicate that roughness does not act as a single universal factor that either increases or decreases ice adhesion strength. Instead, roughness shifts the balance between chemistry-controlled interfacial contributions and morphology-related contributions, and the direction of this shift depends on the coating chemistry. When a coating provides very low ice adhesion strength on the smooth surface, the introduction of micro- and nanotopography can increase the real interaction between ice and solid during freezing and shear removal, which reduces the apparent benefit of the coating. In contrast, for coatings that perform poorly on the smooth substrate, roughness can reduce ice adhesion strength, which indicates that the limiting mechanism on the smooth surface is not directly transferred to the textured case. Therefore, the same increase in roughness can either suppress or improve performance depending on which adhesion contribution is dominant for a given chemistry.

The statistical scatter of the results further shows that roughness influences not only the average ice adhesion strength but also the variability. For some coatings, texturing reduces the separation between chemistries and produces a more clustered response, while for others it increases sample-to-sample scatter. This behavior suggests that roughness introduces additional sensitivity to local conditions, such as spatial non-uniformity of the functional layer within the topography and local variations of the ice–surface contact state formed during freezing. Consequently, paired comparisons provide a practical measure of how robust each chemistry is to increased roughness under the present icing and detachment protocol.

Overall, the results demonstrate that increasing roughness by laser texturing can increase, decrease, or leave nearly unchanged the ice adhesion strength, depending on the applied hydrophobic chemistry. Therefore, roughness cannot be evaluated independently from surface chemistry, and the combined morphology–chemistry interaction must be considered when selecting a coating strategy for icephobic performance.

### 3.4. Comparison with Literature-Reported Data

**Figure 7:**
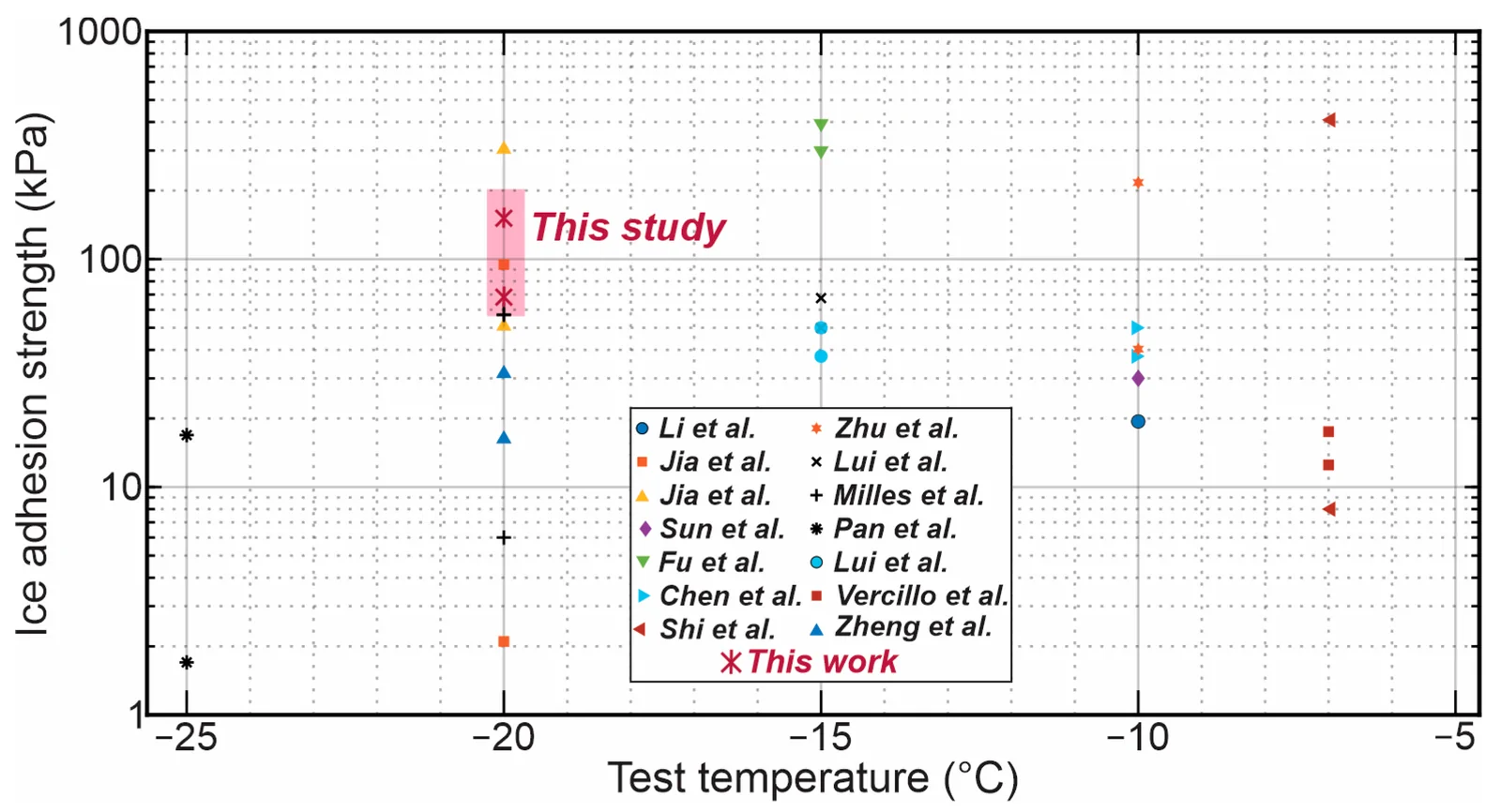
The comparison of the ice adhesion strength data measured in this study with the literature reports [22,25,26,28,31,32,34,35,36,37,38,39,52,53,54].

To put the achieved ice adhesion performance of the developed aluminum surfaces into perspective, we compared the measured ice adhesion strength values with representative values reported in the recent literature for functionalized laser-textured surfaces on different substrate materials. The comparison of our data with those reported in the literature is shown in Figure 7. The literature data presented in Figure 7 are summarized in Table S4, Supplementary Materials, Section S5. The compiled data demonstrates a very broad scatter of reported ice adhesion strength, spanning from approximately 0.01 to 409 kPa over a relatively narrow surface temperature range (−25 to −7 °C). This widespread indicates that the absolute magnitude of ice adhesion strength is strongly influenced not only by the subcooling temperature, but also by differences in surface architecture and, importantly, by variations in the experimental methodology. Therefore, the literature comparison is included primarily to improve the interpretability of our results by placing them within the current performance landscape, rather than to demonstrate record-low ice adhesion. In particular, when the comparison is restricted to aluminum surfaces tested at similar subcooling temperatures, the values obtained in the present study remain within the range reported for functionalized reference and laser-textured surfaces in the literature. In this respect, our measured values on aluminum at −20 °C are ranked within the mid-range of reported values for functionalized reference and functionalized laser-engineered surfaces. In particular, when the comparison is restricted to studies on aluminum surfaces tested around −15 to −20 °C, the literature still reports values ranging from extremely low adhesion to several hundred kPa, and our results fall into a range comparable to multiple reported aluminum datasets while remaining above the best-performing ultra-low adhesion cases. Hence, Figure 7 should be viewed as a contextual benchmark, whereas the key contribution of the present work is the controlled, parameter-focused dataset enabling mechanistic evaluation of how surface modification routes and surface conditions influence ice adhesion strength on aluminum.

The comparison further supports the view that bioinspired superhydrophobicity cannot be assessed by apparent contact angle alone. Literature-reported laser-textured surfaces span a broad range of ice adhesion strengths, which indicates that the effectiveness of a biomimetic texture–chemistry strategy depends strongly on the detailed architecture of the texture, the stability of trapped air or low contact fraction wetting states, and the specific icing protocol.

## 4. Conclusions

This study demonstrates that the anti-icing performance of aluminum surfaces functionalized with different hydrophobic agents depends strongly on the combined effect of coating chemistry and surface morphology. On smooth reference surfaces, with a roughness scale of approximately *R*_a_ = 1.1 μm, the differences in ice adhesion strength were governed primarily by the chemistry of the hydrophobic layer and the resulting interfacial surface free energy. In this case, phosphonic acid-based coatings, especially FDPA, provided the most effective reduction in ice adhesion strength. This can be attributed to their low-surface-energy character and reduced polar interactions at the ice–solid interface.

However, the freezing delay results followed a different trend, with PDMS showing the most favorable response. This indicates that ice adhesion and freezing delay are governed by different mechanisms. Ice adhesion is mainly related to interfacial fracture and ice–solid interactions, whereas freezing delay is more strongly affected by heterogeneous nucleation, coating uniformity, and the polar character of the interface. The poorer freezing delay response of the fatty acid-based coatings suggests that local coating heterogeneity or incomplete screening of the aluminum oxide surface can promote nucleation even when the surface appears hydrophobic.

When the same coatings were applied to laser-textured surfaces, with a roughness scale of approximately *R*_a_ = 5.5 μm, the differences in ice adhesion strength became markedly smaller, and the ranking of coatings no longer followed the behavior observed on the smooth substrates. Although the laser-textured surfaces initially exhibited highly water-repellent wetting states, their freezing delay performance shifted toward shorter times and showed a steeper early temporal decay. This indicates that laser texturing can promote earlier freezing by increasing the available textured area, introducing additional regions for condensation and frost formation, and facilitating local wetting or liquid penetration into the surface features under subzero conditions.

The statistical analysis further confirmed that the anti-icing response cannot be interpreted through coating chemistry or surface morphology alone. Two-way ANOVA showed a statistically significant effect of coating chemistry and a statistically significant morphology–chemistry interaction, whereas the main effect of topography alone was not statistically significant. Overall, the results show that wettability alone is insufficient to predict anti-icing performance. Instead, ice adhesion strength, freezing delay, and water repellency must be interpreted through the coupled influence of surface morphology, wetting-state stability, and coating chemistry.

From a biomimetic design perspective, these results show that transferring the natural principle of hierarchical water repellency to metallic anti-icing surfaces requires more than achieving high apparent contact angles. The laser-textured surfaces reproduced the low-adhesion wetting state associated with natural superhydrophobic interfaces under ambient conditions, but their performance under freezing was limited. Future bioinspired icephobic surfaces should therefore be designed around texture architectures and coating chemistries that preserve trapped air or prevent liquid penetration during cooling, or which provide a slippery interface instead.

## Figures and Tables

**Table 1:** Summary of the literature review on the anti-icing performance of laser-textured surfaces.

Surface or Approach	Substrate and Surface Treatment	Apparent Static Water Contact Angle	Ice Adhesion Strength	Main Findings
Liu et al. [30]	Aluminum; *ns* laser-texturing.	Up to 167°.	Ranged from 300 kPa to 400 kPa.	Laser texturing alone increased ice adhesion when the surface remained hydrophilic. The addition of low-surface-energy coatings reduced ice adhesion substantially.
Liu et al. [31]	Nickel; *fs* laser texturing.	Up to 153°.	Ranged from 25 kPa–100 kPa.	Frosting evolution strongly alters ice adhesion on porous femtosecond laser micro- and nanotextures by changing infiltration, mechanical interlocking, trapped air, and interfacial cracking. Columnar textures are less sensitive to frost and reduce adhesion below one-tenth of untreated surfaces.
Lui et al. [32]	Aluminum; laser texturing.	Up to 156°.	Ranged from 50 kPa–250 kPa.	Ice adhesion on anisotropic grooves depends on the shear direction. A single measurement direction may not fully characterize textured surfaces.
Vercillo et al. [33]	Aluminum and titanium samples. UV *ns* laser texturing.	Up to 169°.	Ranged from 25 kPa–80 kPa.	Single-step laser-induced superhydrophobic surfaces exhibited insufficient durability under aeronautical cycling. Durability improved after subsequent surface functionalization. In glaze icing conditions, adhesion can increase due to enhanced mechanical interlocking within the texture.
Vercillo et al. [34]	Titanium samples; *ns* and *fs* laser texturing.	Up to 168°.	Ranged from 18 kPa–70 kPa.	Ice adhesion depends on the icing regime. Some microtextures promote mechanical interlocking in glaze ice and increase adhesion. Texture scale relative to droplet size remains critical.
Milles et al. [35]	Aluminum; *ns* and *ps* laser texturing.	Up to 171°.	Ranged from 6 kPa–57 kPa.	Optimized laser textures reduced ice adhesion by about 90 percent in wind tunnel icing tests, highlighting the need for realistic testing conditions and careful optimization of texture parameters.
Pan et al. [36]	Copper; *fs* laser texturing.	Up to 162°.	Ranged from 1.7 kPa–207 kPa.	Stabilized, trapped air pockets suppressed interfacial nucleation and enabled extremely low ice adhesion relative to typical superhydrophobic surfaces.
Zheng et al. [26]	Titanium samples; *fs* laser texturing.	Up to 161°.	Ranged from 15 kPa–35 kPa.	Combining laser microstructuring with a dual-layer coating reduced both tensile and shear ice adhesion forces compared to smooth and coating-only surfaces.
Tian et al. [37]	Aluminum; *ns* and fs laser texturing.	Up to 164.	Up to 2 kPa after 1st de-icing cycle; Up to 10 kPa after 30 cycles.	Texture design targeting reduced pinning delivered extremely low initial adhesion and retained low-kPa adhesion after repeated de-icing cycles.
Shu et al. [38]	Copper; *ns* laser texturing.	Up to 160°.	Ranged from 8.2 kPa–410 kPa.	Micro-pit architecture, in combination with a protective low-surface-energy layer, promotes an air layer and reduces interfacial anchoring, thereby improving anti-icing performance and durability.
Chen et al. [29]	Aluminum; Laser texturing and chemical etching.	Up to 161.5°.	Maintained around 10 kPa over 40 icing–de-icing cycles.	Photothermal assistance stabilized low ice adhesion during cycling and enabled rapid melting under illumination.
Chen et al. [28]	Nickel metal surfaces; *fs* laser texturing.	Up to 160°.	Ranged from 14 kPa to193 kPa	Sealed air pockets resisted infiltration, improved de-icing durability, and maintained lower ice adhesion compared to open-pocket designs.
Fu et al. [39]	Aluminium; *ns* laser texturing.	Up to 157°.	Ranged from 350 kPa–2000 kPa.	Higher relative humidity increased ice adhesion through condensation-driven liquid bridges and anchoring.

**Table 2:** Preparation procedures using different hydrophobic agents.

Hydrophobic Agent	Abbreviation	Solution	Preparation Method	Pre-Drying Process	Drying in the Oven
1-phosphonohexadecane	HDPA	3 mM HPDA in ethanol	Immersion coating in the solution for 1 h at 60 °C	/	120 min at 200 °C
3,3,4,4,5,5,6,6,7,7,8,8,9,9,10,10,11,11,12,12,12-henicosafluorododecylphosphonic acid	FDPA	3 mM FDPA in isopropanol	Pipetted droplets onto the surface	Drying at room temperature for 15 min	60 min at 120 °C
Stearic acid	SA	10 mM SA in ethanol	Immersion coating in the solution for 1 h at 60 °C	Washed with ethanol	15 min at 120 °C
Lauric acid	LA	10 mM LA in ethanol	Immersion coating in the solution for 1 h at 60 °C	Washed with ethanol	15 min at 60 °C
Polydimethylsiloxane	PDMS	PDMS in toluene (volumetric ratio: 1: 19)	Chemical vapor deposition for 90 min at 90 °C	/	/

**Table 3:** Measurements of the apparent static contact angle and roll-off angle before and after the ice adhesion tests on reference and laser-textured surfaces functionalized with different hydrophobic agents.

Surface Name	Apparent Static Contact Angle Before Ice Adhesion Strength Tests	Roll-Off Angle Before Ice Adhesion Strength Tests	Apparent Static Contact Angle After Ice Adhesion Strength Tests	Roll-Off Angle After Ice Adhesion Strength Tests
REF	72.5° ± 5.7°	>90°	90.9° ± 3.2°	>90°
REF-HDPA	110.2° ± 5.5°	>90°	107.7° ± 1.7°	* 71.5°; >90°
LT-HDPA	168.8° ± 2.9°	10.9°	155.9° ± 2.4°	* 51°; >90°
REF-FDPA	123.2° ± 4.7°	* 66°; >90°	110.7° ± 18°	* 80.6°; >90°
LT-FDPA	169.8° ± 1.7°	1°	166.2° ± 8.2°	3°
REF-SA	97.8° ± 7.2°	>90°	98.5° ± 5°	>90°
LT-SA	167.8° ± 2.4°	5°	133.7° ± 7.1°	>90°
REF-LA	97.9° ± 7.1°	>90°	91.9° ± 1.8°	>90°
LT-LA	169.3° ± 1.5°	11°	138.5° ± 5.8°	>90°
REF-PDMS	110.5° ± 3.4°	>90°	97.6° ± °4.5°	>90°
LT-PDMS	169° ± 2.4°	4.5°	149.3° ± 16.6°	* 76°; >90°

* The drop rolled off the surface at some spots and remained pinned in others.

## Data Availability

All generated research data is readily available from the manuscript or from the Supplementary Materials.

## Supplementary Materials

The following supporting information can be downloaded at https://www.mdpi.com/article/10.3390/biomimetics11080585/s1: Figure S1: The representative static contact angle images: REF surface (left), REF-HDPA surface (middle) and LT-HDPA surface (right); Figure S2: Cross-sectional morphology and three-dimensional topography of the investigated aluminum surfaces: a) uncoated reference surface (REF), b) HDPA-coated reference surface (REF-HDPA), c) HDPA-coated laser-textured surface (LT-HDPA), and d) three-dimensional optical topography of LT-HDPA; Section S1: Surface analysis; Table S1: Apparent static contact angles measured using diiodomethane; Table S2: Dispersive and polar components of the surface free energy of bare reference aluminum surfaces coated with different hydrophobic agents; Section S2: Surface free energy; Table S3: Kaplan–Meier analysis of the freezing-delay measurements; Section S3: Survival analysis of the freezing-delay measurements; Figure S3: Schematic illustration of the mechanisms governing freezing delay and ice adhesion: a) delayed freezing on the smooth PDMS-coated reference surface, b) earlier freezing on the laser-textured coated surface, c) low ice adhesion on the smooth FDPA-coated reference surface and d) higher ice adhesion on the laser-textured surface due to liquid penetration and mechanical interlocking; Section S4: Mechanistic interpretation of the decoupling between freezing delay and ice adhesion; Table S4: Literature-reported-data; Section S5: Literature-reported data.

## Abbreviations

The following abbreviations are used in this manuscript:

REF	reference surface
LT	laser-textured surface
FDPA	fluorinated phosphonic acid
SA	stearic acid
LA	lauric acid
HDPA	hexadecylphosphonic acid
PDMS	polydimethylsiloxane
SEM	scanning electron microscopy
OWRK	Owens–Wendt–Rabel–Kaelble
