# Supplementary material for: Bioinspired Laser-Textured Aluminum Surfaces for Anti-Icing: Coupled Effects of Hydrophobic Coating Chemistry and Surface Morphology
Source: Biomimetics (Basel). 2026 Aug 17;11(8):585. doi: 10.3390/biomimetics11080585 (PMC13509861; doi:10.3390/biomimetics11080585)
Supplement: Supplementary file 1 [file biomimetics-11-00585-s001.zip › biomimetics-4451321-supplementary.pdf]

Supplementary Materials

Bioinspired Laser-Textured Aluminum Surfaces for Anti-Icing: Coupled Effects of Hydrophobic Coating Chemistry and Surface Morphology

Borut Gregorčič [1,2], Armin Hadžić [1], Jure, Berce [1], Matevž Zupančič [1], Matic Može [1,] and Iztok Golobič [1]*

[1] *University of Ljubljana, Faculty of Mechanical Engineering, Aškerčeva cesta 6, SI-1000 Ljubljana, Slovenia*

[2] *Krka, d. d., Novo mesto, Šmarješka cesta 6, SI-8501 Novo mesto, Slovenia*

* Corresponding author: Matic Može; e-mail: matic.moze@fs.uni-lj.si

Table of Contents

S1. Surface analysis

Figure S1: The representative static contact angle images: REF surface (left), REF-HDPA surface (middle) and LT-HDPA surface (right).

Figure S2: Cross-sectional morphology and three-dimensional topography of the investigated aluminum surfaces: a) uncoated reference surface (REF), b) HDPA-coated reference surface (REF-HDPA), c) HDPA-coated laser-textured surface (LT-HDPA), and d) three-dimensional optical topography of LT-HDPA.

S2. Surface free energy

Table S1: Apparent static contact angles measured using diiodomethane.

Table S2: Dispersive and polar components of the surface free energy of bare reference aluminum surfaces coated with different hydrophobic agents.

S3. Survival analysis of freezing-delay measurements

Table S3: Kaplan–Meier analysis of the freezing-delay measurements.

S4. Mechanistic interpretation of the decoupling between freezing delay and ice adhesion

Figure S3: Schematic illustration of the mechanisms governing freezing delay and ice adhesion: a) delayed freezing on the smooth PDMS-coated reference surface, b) earlier freezing on the laser-textured coated surface, c) low ice adhesion on the smooth FDPA-coated reference surface and d) higher ice adhesion on the laser-textured surface due to liquid penetration and mechanical interlocking.

S5. Literature-reported-data

Table S4: Literature-reported-data.

S6. References

S1. Surface analysis

Representative static contact-angle images of the uncoated reference aluminum surface (REF), the HDPA-coated reference surface (REF-HDPA), and the laser-textured HDPA-coated surface (LT-HDPA) are shown in ***Figure S1***.

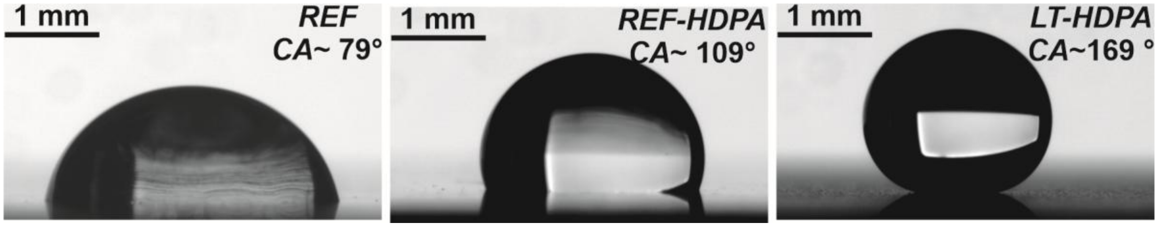

Figure S1: The representative static contact angle images: REF surface (left), REF-HDPA surface (middle) and LT-HDPA surface (right).

To complement the numerical roughness parameters reported in the main manuscript, representative cross-sectional SEM images and three-dimensional optical topography were obtained for selected surfaces. Cross-sectional SEM imaging was performed using a scanning electron microscope for the uncoated reference surface (REF), the HDPA-coated reference surface (REF-HDPA), and the HDPA-coated laser-textured surface (LT-HDPA). The REF and REF-HDPA surfaces exhibit a comparatively flat surface profile, indicating that the thin HDPA functionalization does not introduce a measurable microscale topographical change to the reference aluminum surface. In contrast, the LT-HDPA surface shows a clearly irregular relief associated with the laser-generated hierarchical morphology. Cross-sectional morphology and three-dimensional topography of the investigated aluminum surfaces are shown in ***Figure S2***.

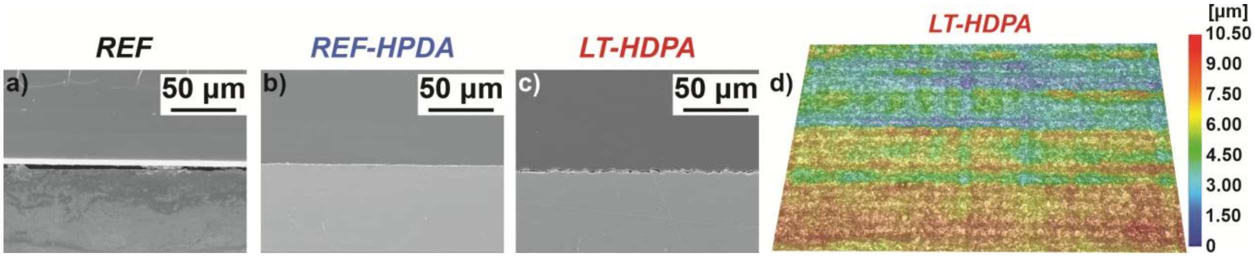

Figure S2: Cross-sectional morphology and three-dimensional topography of the investigated aluminum surfaces: a) uncoated reference surface (REF), b) HDPA-coated reference surface (REF-HDPA), c)

HDPA-coated laser-textured surface (LT-HDPA), and d) three-dimensional optical topography of LT-HDPA.

The three-dimensional surface topography of LT-HDPA was additionally measured using a Keyence optical microscope. The reconstructed height map confirms the presence of spatially distributed ridges, valleys, and overlapping laser-processing tracks, with local height variations extending over the micrometer scale. he combined cross-sectional and three-dimensional characterization therefore confirms that the dominant morphological difference between the reference and laser-textured specimens originates from the laser treatment, whereas the HDPA coating primarily modifies the surface chemistry without substantially changing the underlying microscale morphology.

S2. Surface free energy

The apparent static contact angles measured with diiodomethane on bare reference surfaces functionalized with different hydrophobic coatings are presented in ***Table S1***. The obtained values were used to determine the polar component of the free surface energy of the investigated surfaces.

Table S1: Apparent static contact angles measured using diiodomethane.

Reference surface	FDPA	HDPA	SA	LA	PDMS
Contact angles	82.9 ±2.1	52.9 ±3.4	44.4 ±4.2	39.1 ±4.8	49.6 ±1.6

The dispersive and polar components of the surface free energy, determined from the apparent static contact angles of water and diiodomethane, for bare reference aluminum surfaces coated with different hydrophobic agents are summarized in ***Table S2***.

Table S2: Dispersive and polar components of the surface free energy of bare reference aluminum surfaces coated with different hydrophobic agents.

Hydrophobic coating	Dispersive component (mJ m^{-2})	Polar component (mJ m^{-2})
FDPA	16.03	1.20
HDPA	32.64	1.43
SA	37.33	1.26
LA	40.09	1.29
PDMS	34.47	1.43

S3. Survival analysis of freezing-delay measurements

To account for droplets that did not freeze within the prescribed observation period, the freezing-delay data were additionally evaluated using Kaplan–Meier survival analysis. The initiation of surface cooling was defined as t=0. Droplets denoted as not iced after 3600 s were treated as right-censored at 3600 s. For the restricted analysis, any freezing event recorded after 3600 s was also truncated at the analysis horizon.

The Kaplan–Meier median freezing time was defined as the earliest time at which the estimated probability of remaining unfrozen decreased to 0.5. When more than 50% of the droplets remained unfrozen at the end of the observation period, the median freezing time could not be estimated and was reported as not reached. The restricted mean survival time up to 3600 s, denoted as RMST_{3600}, was calculated as:

$$\mathrm{RMST}_{3600} = \int_0^{3600} \hat{S}\,(t)\,\mathrm{d}t, \tag{S1}$$

where $\hat{S}(t)$ is the Kaplan–Meier estimate of the probability that a droplet remains unfrozen at time t. Unlike an arithmetic mean calculated only from droplets that froze, RMST_{3600} incorporates the complete observation period for both frozen and right-censored droplets.

The arithmetic mean freezing times reported in the main manuscript describe only the droplets that froze during the experiment and are retained as descriptive values. The censoring-aware median freezing times and RMST_{3600} values are summarized in ***Table S3***.

Table S3: Kaplan–Meier analysis of the freezing-delay measurements.

**Surface condition**	**Total droplets, (n)**	**Frozen by 3600 s, (n) (%)**	**Unfrozen at 3600 s, (n) (%)**	**Kaplan–Meier median (s)**	$RMST_{3600}$ (s)
REF	32	32 (100%)	0 (0%)	508.8	515.2
REF–PDMS	32	14 (43.8%)	18 (56.3%)	NR	2421.7
REF–HDPA	32	32 (100%)	0 (0%)	46.0	162.8
REF–FDPA	32	27 (84.4%)	5 (15.6%)	65.0	737.6
REF–LA	32	30 (93.8%)	2 (6.3%)	41.0	363.3
REF–SA	32	32 (100%)	0 (0%)	45.0	47.1
LT–PDMS	32	30 (93.8%)	2 (6.3%)	138.0	646.3
LT–HDPA	32	32 (100%)	0 (0%)	59.0	242.6
LT–FDPA	32	32 (100%)	0 (0%)	47.0	62.9
LT–LA	32	32 (100%)	0 (0%)	196.0	355.1
LT–SA	32	31 (96.9%)	1 (3.1%)	60.0	472.2

For LT–PDMS, one droplet froze at 3637 s. Because $RSMT_{3600}$ was restricted to the predefined 3600 sanalysis horizon, this observation was treated as unfrozen at 3600 S for the calculation presented in ***Table S3***. Separate overall log-rank tests showed statistically significant differences among the reference-surface conditions X^2=90.96, *df*=5 and *p*<0.001 and among the laser-textured conditions X^2=35.29, *df*=4 and *p*<0.001. The survival analysis particularly strengthens the interpretation of the REF–PDMS surface. Only 14 of 32 droplets froze during the 3600 sobservation period, while 18 droplets remained unfrozen. Consequently, the Kaplan–Meier survival probability remained above 0.5 and the median freezing time was not reached. Its $RMST_{3600}$ of 2421.7 s was also substantially higher than that of the other investigated conditions, confirming the pronounced freezing-delay response of the smooth PDMS-coated surface.

S4. Mechanistic interpretation of the decoupling between freezing delay and ice adhesion

Figure S3 schematically summarizes the distinct mechanisms governing freezing delay and ice adhesion in the present study. For example, on the smooth PDMS-coated reference surface, the relatively uniform low-polarity interface reduces the effectiveness of heterogeneous nucleation sites, which is consistent with the longest measured freezing delay. In contrast, on laser-textured coated surfaces, hierarchical morphology introduces asperity edges, cavities, and a larger effective surface area.

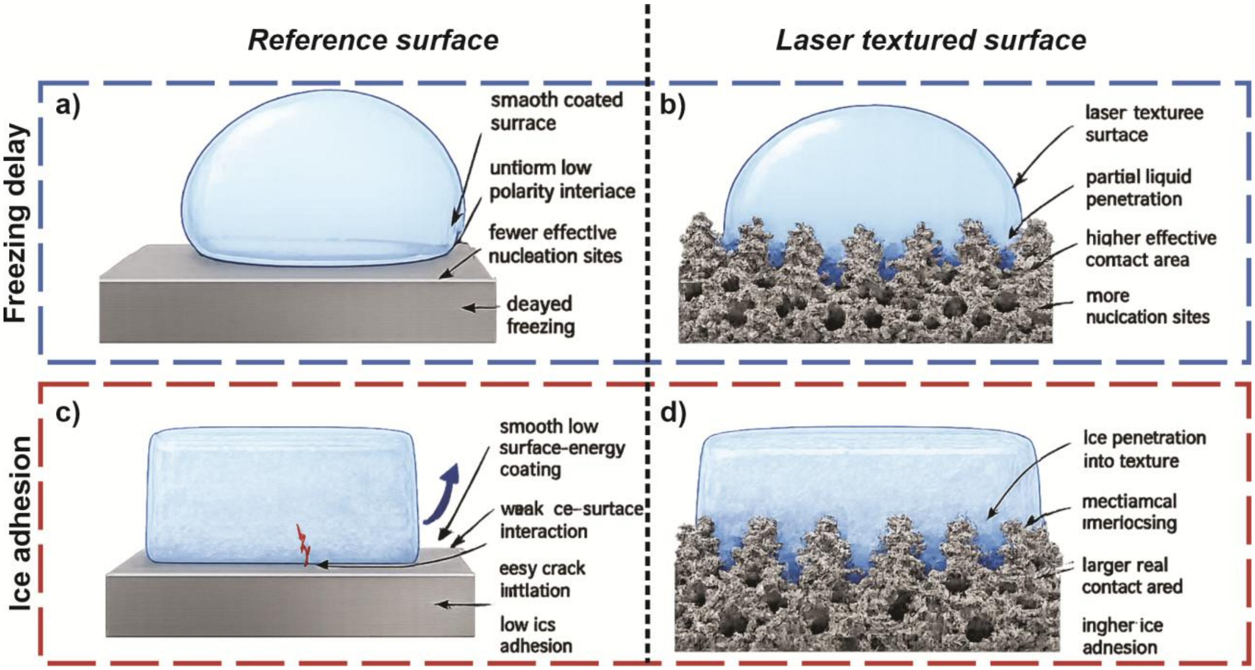

Figure S3: Schematic illustration of the mechanisms governing freezing delay and ice adhesion: a) delayed freezing on the smooth PDMS-coated reference surface, b) earlier freezing on the laser-textured coated surface, c) low ice adhesion on the smooth FDPA-coated reference surface and d) higher ice adhesion on the laser-textured surface due to liquid penetration and mechanical interlocking.

During cooling, partial liquid penetration into the laser-generated texture can occur, increasing the effective liquid–solid contact area and activating additional heterogeneous nucleation sites, thereby promoting earlier freezing. Ice adhesion is governed by a different interfacial state, namely the fully formed ice–surface interface. For instance, on the smooth FDPA-coated reference surface,

the low-surface-energy chemistry weakens interfacial ice–solid interactions and facilitates crack initiation, resulting in low ice adhesion. On laser-textured surfaces, however, penetration and freezing of water within the texture can increase the real contact area and promote mechanical interlocking of ice with the surface features, leading to higher adhesion. The schematic therefore illustrates why a surface exhibiting longer freezing delay does not necessarily exhibit lower ice adhesion, which is fully consistent with the discussion in the main manuscript.

S5. Literature-reported-data

The literature data used for comparison in Figure 7 is summarized in ***Table S4***.

Table S4: Literature-reported-data.

**Authors**	**Surface material**	**Temperature (°C)**	**Ice adhesion (kPa)**
Li et al. [1]	Stainless steal	-10	19.4
Jia et al. [2]	Silicon rubber	-20	94.7
		-20	2.1
Jia et al. [3]	Silicon rubber	-20	298.9
		-20	50
Sun et al. [4]	Aluminium	-10	30
Fu et al. [5]	Aluminium	-15	304
		-15	400
Chen et al. [6]	Aluminium	-10	37.5
		-10	50
Shu et al. [7]	Copper	-7	8.12
		-7	408.6
Tian et al. [8]	Aluminium	-20	0.01
		-20	0.07
Zhu et al. [9]	Epoxy resin based	-10	40.3
		-10	217
Liu et al. [10]	Aluminium	-15	50
		-15	67.5
Milles et al. [11]	Aluminium	-20	6
		-20	57
Pan et al. [12]	Copper	-25	1.7
		-25	16.9
Lui et al. [13]	Aluminium	-15	37.5
		-15	50
Zheng et al. [14]	Titanium	-7	12.5
		-7	17.5
Vercillo et al. [15]	Titanium	-20	16
		-20	31
Our study	Aluminium	-20	68.9
		-20	152.1

S6. References

[1] Z. Li, S. Li, X. Su, Z. He, J. Gu, Y. Diao, J. Xu, B. Guo, Anti-icing Performance of Superhydrophobic Surfaces with Periodic Micro-nano Structures Directly Induced by Femtosecond Laser, 2400828 (2025) 1–11. https://doi.org/10.1002/admi.202400828.

[2] Y. Jia, A. Li, J. Wang, X. Wang, F. Zhang, Q. Guo, Durable Ceramicization-Induced Uncoated Superhydrophobic Surfaces for Anti-Icing Propeller Edges, 07777 (2025) 1–9. https://doi.org/10.1002/adma.202507777.

[3] Y. Jia, A. Li, Z. Xu, K. Qi, J. Zhong, F. Zhang, Q. Guo, Laser-induced ash-deposited silicone rubber surfaces with synergistic micro / nanostructures for enhanced superhydrophobicity and anti- / de-icing performance, Chem. Eng. J. 523 (2025) 168252. https://doi.org/10.1016/j.cej.2025.168252.

[4] J. Sun, Z. Cao, Y. Nie, J. Peng, Y. Ma, T. Rao, Y. Wang, G. Wang, P. Wang, Icephobic armored ceramic via hierarchical microarchitecture engineering : Achieving damage-tolerant surfaces with sustained dynamic anti-icing performance, Results Chem. 16 (2025) 102393. https://doi.org/10.1016/j.rechem.2025.102393.

[5] D. Fu, H. Zheng, W. Sheng, X. Hao, X. Zhang, S. Chang, An experimental study on the influence of humidity on ice adhesion strength on superhydrophobic surfaces with microstructures, Appl. Therm. Eng. 244 (2024) 122732. https://doi.org/10.1016/j.applthermaleng.2024.122732.

[6] Z. Chen, L. Song, Y. Wang, H. Tao, Z. Liu, T. Wang, F. Ye, Y. He, J. Lin, Air pocket-optimization strategy for micro / nanostructures fabricated by femtosecond laser technology for anti-icing performance improvement, Appl. Surf. Sci. 655 (2024) 159454. https://doi.org/10.1016/j.apsusc.2024.159454.

[7] Y. Shu, X. Lu, W. Lu, W. Su, Y. Wu, H. Wei, Mechanically robust superhydrophobic copper surface with self-cleaning , anti-icing , and corrosion resistance, Surf. Coat. Technol. 455 (2023) 129216. https://doi.org/10.1016/j.surfcoat.2022.129216.

[8] Z. Tian, P. Fan, D. Zhu, L. Wang, H. Zhao, C. Chen, R. Peng, D. Li, H. Zhang, M. Zhong, Anti-ice-pinning superhydrophobic surfaces for extremely low ice adhesion, Chem. Eng. J. 473 (2023) 145382. https://doi.org/10.1016/j.cej.2023.145382.

[9] Z. Zhu, P. Wu, S. Juodkazis, J. Wang, S. Yao, J. Yao, W. Zhang, Superhydrophobic and Anti-Icing Surface by Femtosecond Laser Direct Writing, 2300575 (2023) 1–11. https://doi.org/10.1002/adem.202300575.

[10] Z. Liu, F. Ye, H. Tao, J. Lin, Effects of frost formation on the ice adhesion of micro-nano structure metal surface by femtosecond laser, J. Colloid Interface Sci. 603 (2021) 233–242. https://doi.org/10.1016/j.jcis.2021.06.105.

[11] S. Milles, V. Vercillo, S. Alamri, A.I. Aguilar-Morales, T. Kunze, E. Bonaccurso, A.F. Lasagni, Icephobic performance of multi-scale laser-textured aluminum surfaces for aeronautic applications, Nanomaterials. 11 (2021) 1–17. https://doi.org/10.3390/nano11010135.

[12] R. Pan, H. Zhang, M. Zhong, Triple-Scale Superhydrophobic Surface with Excellent Anti-Icing and Icephobic Performance via Ultrafast Laser Hybrid Fabrication, ACS Appl. Mater. Interfaces. 13 (2021) 1743–1753. https://doi.org/10.1021/acsami.0c16259.

[13] Z. Liu, H. Tao, J. Lin, Anisotropic Ice Adhesion of Micro-Nano-Structured Metal Surface by a Femtosecond Laser, (2021). https://doi.org/10.1021/acs.langmuir.1c01459.

[14] H. Zheng, S. Chang, G. Ma, S. Wang, Anti-icing performance of superhydrophobic surface fabricated by femtosecond laser composited dual-layers coating, Energy Build. 223 (2020) 110175. https://doi.org/10.1016/j.enbuild.2020.110175.

[15] V. Vercillo, S. Tonnicchia, J.M. Romano, A. García-Girón, A.I. Aguilar-Morales, S. Alamri, S.S. Dimov, T. Kunze, A.F. Lasagni, E. Bonaccurso, Design Rules for Laser-Treated Icephobic Metallic Surfaces for Aeronautic Applications, Adv. Funct. Mater. 30 (2020). https://doi.org/10.1002/adfm.201910268.
